# Proteome Profile of Myelin in the Zebrafish Brain

**DOI:** 10.3389/fcell.2021.640169

**Published:** 2021-04-08

**Authors:** Sophie B. Siems, Olaf Jahn, Laura J. Hoodless, Ramona B. Jung, Dörte Hesse, Wiebke Möbius, Tim Czopka, Hauke B. Werner

**Affiliations:** ^1^Department of Neurogenetics, Max Planck Institute for Experimental Medicine, Göttingen, Germany; ^2^Proteomics Group, Max Planck Institute for Experimental Medicine, Göttingen, Germany; ^3^Centre for Clinical Brain Sciences, The University of Edinburgh, Edinburgh, United Kingdom; ^4^Electron Microscopy Core Unit, Max Planck Institute for Experimental Medicine, Göttingen, Germany

**Keywords:** oligodendrocyte, myelin proteome, myelin evolution, label-free proteomics, MBP, MPZ, CD59, zebrafish *Danio rerio*

## Abstract

The velocity of nerve conduction along vertebrate axons depends on their ensheathment with myelin. Myelin membranes comprise specialized proteins well characterized in mice. Much less is known about the protein composition of myelin in non-mammalian species. Here, we assess the proteome of myelin biochemically purified from the brains of adult zebrafish (*Danio rerio*), considering its increasing popularity as model organism for myelin biology. Combining gel-based and gel-free proteomic approaches, we identified > 1,000 proteins in purified zebrafish myelin, including all known constituents. By mass spectrometric quantification, the predominant Ig-CAM myelin protein zero (MPZ/P0), myelin basic protein (MBP), and the short-chain dehydrogenase 36K constitute 12%, 8%, and 6% of the total myelin protein, respectively. Comparison with previously established mRNA-abundance profiles shows that expression of many myelin-related transcripts coincides with the maturation of zebrafish oligodendrocytes. Zebrafish myelin comprises several proteins that are not present in mice, including 36K, CLDNK, and ZWI. However, a surprisingly large number of ortholog proteins is present in myelin of both species, indicating partial evolutionary preservation of its constituents. Yet, the relative abundance of CNS myelin proteins can differ markedly as exemplified by the complement inhibitor CD59 that constitutes 5% of the total zebrafish myelin protein but is a low-abundant myelin component in mice. Using novel transgenic reporter constructs and cryo-immuno electron microscopy, we confirm the incorporation of CD59 into myelin sheaths. These data provide the first proteome resource of zebrafish CNS myelin and demonstrate both similarities and heterogeneity of myelin composition between teleost fish and rodents.

## Introduction

Myelination of vertebrate axons accelerates nerve conduction by facilitating saltatory impulse propagation (Tasaki, 1939; Hartline and Colman, 2007). Indeed, myelin has evolved to enable the normal motor, sensory, and cognitive capabilities of vertebrates, as illustrated by their decline and the severe clinical presentations of human patients with myelin-related disorders (Stadelmann et al., 2019; Wolf et al., 2020). The typical multimembrane structure of compact myelin necessitates specialized proteins that mediate the apposition and adhesion of adjacent membrane layers (Nave and Werner, 2014; Snaidero and Simons, 2017). It has long been thought that the complexity of myelin protein composition is very low. This view was founded on studies in the 1970s, in which myelin was biochemically purified from nervous tissue and separated by polyacrylamide gel electrophoresis (PAGE). Upon protein staining using Coomassie Blue or other staining dyes, myelin purified from the brains of mammalian, avian, or reptilian species displayed only four to five bands (Zanetta et al., 1977; Quarles et al., 1978; Franz et al., 1981; Jeserich, 1983), which we now know represent one or several splice isoforms each of the transmembrane-tetraspan proteolipid protein (PLP), the cytosolic adhesion protein myelin basic protein (MBP), and the enzyme cyclic nucleotide phosphodiesterase (CNP, previously termed Wolfgram protein). Notably, the band pattern of central nervous system (CNS) myelin of several species of bony fish differed markedly from that of tetrapod myelin with respect to the number of bands and their molecular weight (Franz et al., 1981; Jeserich, 1983; Waehneldt et al., 1986; Kirschner et al., 1989). Indeed, the major bands of fish myelin were constituted by one or several splice isoforms of MBP, one or two variants of the Ig-CAM myelin protein zero (MPZ, previously termed intermediate proteins IP1 and IP2) and a protein migrating at the molecular weight of 36 kDa (termed 36K). Together, these results have indicated an evolutionary shift of myelin protein composition at the transition from fish to tetrapods (Waehneldt, 1990; Yoshida and Colman, 1996; Schweigreiter et al., 2006; Yin et al., 2006).

The method to enrich myelin as a lightweight membrane fraction from nervous tissue by an established protocol involving sucrose density gradient centrifugation and osmotic shocks has been adapted only slightly since the 1970s (Norton and Poduslo, 1973; Erwig et al., 2019a), reflecting that the protocol is suited to purify myelin membranes to a degree of about 95% (De Monasterio-Schrader et al., 2012). However, the techniques of gel staining and protein identification have markedly improved since. Indeed, protein staining using Coomassie Brilliant Blue (CBB250) upon separation of myelin on contemporary gradient gels yields numerous distinct bands (Morris et al., 2004; Avila et al., 2007; De Monasterio-Schrader et al., 2012), the major constituents of which can be identified by mass spectrometric methods. For example, this strategy allowed identification of several constituents of zebrafish myelin, i.e., of the 36K protein as a short-chain dehydrogenase/reductase [36K, also termed DHRS12 (Morris et al., 2004)], Zwilling proteins (ZWIa and ZWIb) (Schaefer and Brösamle, 2009), and two MBP paralogs [MBPa, MBPb (Nawaz et al., 2013)].

More recently, the advent of label-free shotgun proteomic approaches (reviewed in Neilson et al., 2011) has allowed circumvention of any possible bias introduced by gel separation or staining of proteins. Importantly, these techniques facilitate simultaneous identification and quantification of proteins without the need of introducing costly stable isotope labels. With the aim of routinely exploring the relative abundance of hundreds of proteins in myelin, we chose a peptide intensity-based quantification approach with a data-independent acquisition (DIA) strategy that relies on collecting data in an alternating low and elevated energy mode (referred to as MS^E^) and on ion mobility spectrometry to deconvolute spectral complexity (referred to as UDMS^E^; reviewed in Distler et al., 2014b). As recently described in detail (Siems et al., 2020), these approaches have allowed us to establish the proteome profiles of myelin in both the CNS and the peripheral nervous system (PNS) of mice (Jahn et al., 2020; Siems et al., 2020). Together, myelin proteome analysis has contributed to the insight that the protein composition of myelin is considerably more complex than initially thought. Considering that the relative abundance of known myelin proteins displays a dynamic range of more than four orders of magnitude, we now know that exceptionally abundant myelin proteins have overshadowed low-abundant constituents in early gel-based approaches.

The zebrafish has become a frequently used model organism to study cellular and molecular mechanisms in the central nervous system, including myelination (Pogoda et al., 2006; Lyons and Talbot, 2015; Ackerman and Monk, 2016; Czopka, 2016) and oligodendrocyte differentiation (Ravanelli et al., 2018; Marisca et al., 2020). Notwithstanding the increasing relevance of zebrafish in assessing the pathomechanisms of human myelin-related diseases (Pérez-Rius et al., 2019; Keefe et al., 2020; Tsata et al., 2020), proteomic approaches to zebrafish myelin have fallen behind evolving technical standards. For example, no study is available thus far that systematically approaches the entire zebrafish CNS myelin proteome.

Together with trout, goldfish, salmon, tilapia, eel, and about 30,000 other species, zebrafish belong to the largest vertebrate clade, a group of bony fish termed teleosts (Ravi and Venkatesh, 2018). Much of the considerable morphological and physiological diversity among teleost fish has been attributed to a comparatively fast rate of protein evolution after an event of whole-genome duplication at the root of teleosts, upon which many duplicated genes were retained as paralogs (also termed ohnologs). We note that other teleost species besides zebrafish may also emerge as valuable models in myelin research, including medaka (Dodo et al., 2020). Yet, compared to other fish species, most systematic molecular data are presently available for zebrafish, including genome, transcriptome, and reference proteome datasets. The phenotypic diversity of teleosts and the large number of paralog genes notwithstanding, zebrafish are thus not only suited as model systems in myelin biology with medical relevance, but also to represent fish in evolutionary cross-species comparisons.

Here, we considered that systematically approaching the protein composition of CNS myelin in zebrafish is beneficial for both using zebrafish as a model for myelin-related disorders as well as systematically approaching the evolution of myelin protein composition. We thus assessed CNS myelin purified from adult zebrafish by proteome analysis using a combination of gel-based and gel-free proteomic approaches.

## Materials and Methods

### Animals

#### Zebrafish

The following zebrafish lines and strains were used: AB wild type, *Tg(olig1:nls-mApple)* (Marisca et al., 2020) and *Tg(mbp:nls-GFP)* (Karttunen et al., 2017). Zebrafish were kept at 28.5°C with a 14-h/10-h light–dark cycle, in accordance with United Kingdom Home Office regulations (project license PP5258250). Dissection of adult zebrafish for myelin purification was previously reported (Nawaz et al., 2013).

#### Mice

For myelin purification, we used male c57Bl/6N mice at the ages of postnatal day 18 (P18), P75, and 6 months. For cryo-immuno electron microscopy, we used male c57BL/6N mice at P75. Mice were bred and maintained in the animal facility of the Max Planck Institute for Experimental Medicine; they were sacrificed by cervical dislocation. For sacrificing mice to subsequently dissect tissue, all regulations given in the German Animal Protection Law (TierSchG §4) were followed. Sacrificing of vertebrates is not an experiment on animals according to §7 Abs. 2 Satz 3 TierSchG; therefore, no specific authorization or notification was required for the current work.

### Myelin Purification and Gel Staining

Purification of a lightweight membrane fraction enriched for myelin from the brains and optic nerves of three pools of adult zebrafish (length 2–3 cm; age 4–12 months; 50 fish/pool) by sucrose density centrifugation and osmotic shocks was previously reported (Nawaz et al., 2013). Electron microscopy of the zebrafish myelin-enriched fraction was performed as previously reported for myelin purified from mouse brains (Werner et al., 2007). Purification of myelin from the brains of mice at the indicated ages for immunoblot analysis (in Figure 5) was performed as previously described (Patzig et al., 2016a; Erwig et al., 2019a). Protein concentrations were determined using the DC Protein Assay Kit (Bio-Rad). Gel electrophoresis was performed as detailed below and proteins were visualized using Coomassie Brilliant Blue G-250 or by silver staining as previously described (Schardt et al., 2009; Erwig et al., 2019a).

### Immunoblot

For immunoblot analysis, samples were diluted in 1 × SDS sample buffer and dithiothreitol. To deglycosylate proteins, samples were incubated with Endoglycosidase H (BioLabs, Cat# P0703S), 10× Glycoprotein Denaturing Buffer (BioLabs, Cat# B1720S), and ddH_2_O for 50 min at 38°C. After adding loading buffer, the samples were heated for 10 min at 40°C before loading onto gels. In total, 15 μg per sample was loaded onto 15% acrylamide gels and proteins were separated by SDS-PAGE (180V) using Mini-PROTEAN Handcast system (Bio-Rad, Munich, Germany). Protein transfer was performed as previously described (Patzig et al., 2016b; Siems et al., 2020). Primary antibodies were specific for CD59 (Invitrogen, Cat# PA5-97565; 1:500) and cyclic nucleotide phosphodiesterase (CNP; Sigma, Cat# C5922; 1:1000). HRP-coupled secondary anti-mouse or -rabbit antibodies were from dianova (1:10,000). Immunoblots were developed using the enhanced chemiluminescent detection kit (ECL; Western Lightning^®^ Plus-ECL); signals were detected using the Intas ChemoCam system (Intas Science Imaging, Göttingen, Germany).

### Gel-Based Proteome Analysis of Myelin

Separation of myelin proteins by gel electrophoresis was performed on pre-cast 8–16% Tris-glycine gradient gels (TG PRiME, Serva) as described in detail (Erwig et al., 2019a; Jahn et al., 2020). For systematic proteome analysis from entire gel lanes, 5 μg of protein was loaded before (pre-wash) or after (post-wash) subjecting myelin to sequential washing/centrifugation cycles of high salt and high pH as previously described (Werner et al., 2007; Jahn et al., 2013). Complete lanes were sliced into uniform pieces (the exact number of pieces depending on the swelling status of the gel) and subjected to automated tryptic in-gel digest of proteins (Schmidt et al., 2013) followed by identification of proteins by LC-MS as previously described (Ott et al., 2015).

### Gel-Free, Label-Free Identification and Quantification of Myelin Proteins

In-solution digestion of myelin proteins was performed according to a protocol of automated filter-aided sample preparation (FASP) (Erwig et al., 2019a) originally described by Manza et al. (2005). LC-MS analysis was performed by two MS^E^-type DIA mass spectrometry approaches as recently described for mouse CNS (Jahn et al., 2020) and PNS myelin (Siems et al., 2020). In brief, protein fractions equivalent to 10 μg of myelin protein from three biological replicate pools were dissolved in lysis buffer (1% ASB-14, 7 M urea, 2 M thiourea, 10 mM DTT, and 0.1 M Tris, pH 8.5) and subjected to CHAPS-based FASP in centrifugal filter units (30 kDa MWCO, Merck Millipore). After removing the detergents, protein alkylation with iodoacetamide, and buffer exchange to digestion buffer [50 mM ammonium bicarbonate (ABC), 10% acetonitrile], proteins were digested with 400 ng trypsin at 37°C overnight. Tryptic peptides were recovered by centrifugation and extracted with 40 μl of 50 mM ABC and 40 μl of 1% trifluoroacetic acid (TFA). Combined flow-throughs were subjected to LC-MS analysis. For quantification using the TOP3 approach (Silva et al., 2006; Ahrné et al., 2013), aliquots were spiked with 10 fmol/μl of a tryptic digest of yeast enolase-1 (Waters Corporation).

Peptide separation by nanoscale reversed-phase UPLC was performed on a nanoAcquity system (Waters Corporation) as previously described (Jahn et al., 2020; Siems et al., 2020). Mass spectrometric analysis on a quadrupole time-of-flight mass spectrometer with ion mobility option (Synapt G2-S, Waters Corporation) was performed in the ion mobility-enhanced DIA mode with drift time-specific collision energies referred to as UDMS^E^ (Distler et al., 2014a). As established previously for proteome analysis of purified mouse myelin (Jahn et al., 2020; Siems et al., 2020), samples were re-run in a data acquisition mode without ion mobility separation of peptides (referred to as MS^E^) to ensure the correct quantification of exceptionally abundant myelin proteins.

Processing of LC-MS data and database searching for protein identification was performed using Waters ProteinLynx Global Server (PLGS) version 3.0.3 with published settings (Jahn et al., 2020; Siems et al., 2020). Aiming at a reasonably sized search space with preferably complete, but non-redundant protein annotations, we used a downloadable version of the UniProtKB *Danio rerio* reference proteome containing a single representative protein sequence per gene with reviewed (Swiss-Prot) entries where possible (release 2020_01, proteome ID UP000000437, 25701 entries). Based on prior knowledge, this database was curated by replacing the MBPb entry (accession F8W4C1 instead of E7EYA2) and by adding the entries for NFASCb (A0A4P8NJ80), ZWIa (B9UZF4), and ZWIb (B9UZF5). This FASTA file was used to compile a custom database by further adding the sequence information for yeast enolase-1 (P00924) and porcine trypsin (P00761) and by appending the reversed sequence of each entry to enable the determination of false discovery rate (FDR). This curated database is part of the proteomics data deposited in a publicly available repository (see below).

For post-identification analysis including TOP3 quantification of proteins, the software ISOQuant (Distler et al., 2014a; Kuharev et al., 2015) was used (freely available at www.isoquant.net) and proteins were quantified as parts per million (ppm) as previously described (Jahn et al., 2020; Siems et al., 2020), i.e., the relative amount (w/w) of each protein in respect to the sum over all detected proteins. FDR for both peptides and proteins was set to 1% threshold. Only proteins represented by at least two peptides (one of which unique) and quantified in at least two out of three replicates were reported. The mass spectrometry proteomics data have been deposited to the ProteomeXchange Consortium via the PRIDE (Perez-Riverol et al., 2019) partner repository with dataset identifier PXD023037.

### Generation of Transgenesis Constructs

The constructs pME_sigpep-EYFP-nostop, pME_mScarlet-CAAX, p3E_CD59-pA, pTol2_mbp:EYFP-CD59, and pTol2_mbp:mScarletCAAX were newly generated for this study. All primer sequences are given in Table 1. The entry clone p5E_mbp was previously reported (Almeida et al., 2011).

**TABLE 1:** Primers used for cloning.

Primer	Sequence
**pME_sigpep-YFP-nostop**	
attB1_sigpep_F	GGGGACAAGTTTGTACAAAAAAGCAGG CTGCCACCATGAAAGCTTCTGTCGGAG
Sigpep_YFP_F	ATGAAAGCTTCTGTCGGAGTGTGTGTGGT TTTCGTGCTGGCTCTGCTGGGGCTTGGTT CTGCCatggtgagcaagggcgag
attB2R_YFP_nostop_R	GGGGACCACTTTGTACAAGAAAGCTGGG Tccttgtacagctcgtccatg
**p3E_CD59pA**	
*Cla*I_CD59_F	GACTATCGATATTAAATGTTACAATTGTA AGGAC
XbaI_CD59_R	GATCTAGATTAGAAAACACCCCACCAG
attB2_CD59_F	GGGGACAGCTTTCTTGTACAAAGTGGTAA TTAAATGTTACAATTGTAAGGAC
attB3R_pA_R	GGGGACAACTTTGTATAATAAAGTTG
**pME_mScarlet-CAAX**	
attB1F_mScarlet-CAAX_F	GGGGACAAGTTTGTACAAAAAAG
mScarlet-CAAX_R	TCAGGAGAGCACACACTTGCAGCTCATG CAGCCGGGGCCACTCTCATCAGGAGGGT TCAGCTTCTTGTACAGCTCGTCCATG
attB2R_CAAX-mScarlet_R	TCAGGAGAGCACACACTTGCAGCTCATGCA GCCGGGGCCACTCTCATCAGGAGGGTT CAGCTTCTTGTACAGCTCGTCCATG

The middle entry clone pME_sigpep-EYFP-nostop was generated in a two-step PCR. First, the CD59 signal peptide coding for import to the secretory pathway (bases 1–64) was attached to EYFP by PCR on a EYFP-containing plasmid as template using the sigpep YFP_F primer and the attB2R_YFP_nostop_R. The second PCR was performed using the product of the first PCR as template with primers attB1_sigpep_F and attB2R_YFP_nostop_R. The resulting was recombination-cloned into pDONR221 using BP Clonase (Invitrogen).

The middle entry clone pME_mScarlet-CAAX was generated in a similar two-step PCR strategy to attach the CAAX membrane targeting motif with attB1 and attB2R recombination sites to mScarlet, which was amplified from an mScarlet -containing template plasmid (see Table 1). The PCR product was then recombination cloned into pDONR221 using BP clonase (Invitrogen).

To generate the 3′ entry clone p3E_CD59-pA, CD59 (bases 64–357 of ENSDART00000126737.4), lacking the signal peptide, was amplified using the primers *Cla*I_CD59_F and XbaI_CD59_R from cDNA from 5 days post fertilization (dpf) wild-type zebrafish and cloned into pCS2 + using *Cla*I/Xbal restriction sites. Then, CD59pA was PCR amplified from this plasmid using the primers attB2_CD59_F and attB3R_pA_R and cloned into pDONR_P2R-P3 using BP Clonase.

The transgenic expression constructs pTol2_mbp:mScarlet-CAAX and pTol2_mbp:EYFP-CD59 were generated by multisite LR recombination reactions using the abovementioned and additional entry clones from the Tol2 kit (Kwan et al., 2007).

### DNA Microinjection and Mounting of Embryos for Live Cell Microscopy

Fertilized wild-type zebrafish eggs were pressure-injected with 1 nl of an injection solution containing 10 ng/μl of mbp:mScarlet-CAAX DNA, 10 ng/μl of mbp:EYFP-CD59 DNA, and 50 ng/μl of Tol2 transposase mRNA and 10% phenol red. Zebrafish embryos were anesthetized using 0.2 mg/ml of tricaine mesylate (Sigma Aldrich). For confocal imaging, embryos were mounted laterally in 1% ultrapure low melting point agarose (Invitrogen) on a glass coverslip as previously described (Vagionitis and Czopka, 2018).

### Tissue Preparation and Cryosectioning

Zebrafish embryos were euthanized 5 or 6 dpf with 4 mg/ml tricaine mesylate, fixed overnight at 4°C in 4% paraformaldehyde. Fixed animals were cryoprotected for 3 days in serially increasing sucrose concentrations (10, 20, and 30%) and then embedded in Tissue Tek. Transverse sections of 14–16 μm thickness were cut using a Leica CM1850 UV cryostat.

### *In situ* Hybridization and Immunohistochemistry

RNA probes were used to detect zebrafish *cd59* (ENSDARG00000090615, ACD probe catalog code: 561561-C2). We used the RNAScope Multiplex Fluorescent V2 kit (ACD) on cryosections according to the manufacturer’s protocol for fixed-frozen samples. Signals were detected with TSA-conjugated Opal Dyes (Perkin Elmer), as detailed in Table 2. Following RNA hybridization, immunohistochemistry was performed to detect transgenically expressed fluorescent proteins. Sections were blocked for 1.5 h at room temperature in PBS containing 0.1% Tween 20, 10% FCS, 0.1% BSA, and 3% normal goat serum. Primary antibodies were incubated at 4°C overnight in blocking solution. Sections were washed three times in PBS (with 0.1% Tween 20) and incubated with Alexa Fluor (AF)-conjugated secondary antibodies. Antibodies used are listed in Table 2. Stained sections were washed twice in PBS with 0.1% Tween 20, once in PBS, and mounted with ProLong Diamond Antifade mountant with DAPI (Thermo Fisher Scientific).

**TABLE 2:** Immunohistochemistry.

Antibody/Dye	Concentration	Manufacturer	Cat No.
Anti-GFP	1 in 2000	Abcam ab13970	GR236651-23
Anti-DsRed (for mApple)	1 in 1000	Takara/Clontech632496	PK0495
Goat anti-chicken AF 488	1 in 1000	Invitrogen A-11039	1899514
Goat anti-rabbit AF 555	1 in 1000	Invitrogen A-21428	1903133
Opal 650	1 in 1000	Perkin Elmer	FP1496A

### Microscopy

Twelve-bit confocal images were acquired on a Leica TCS SP8 laser scanning microscope. A 488-nm wavelength was used to excite Alexa Fluor 488, a 514-nm wavelength was used to excite EYFP, a 561-nm wavelength was used to excite mScarlet or Alexa Fluor 555, and a 633-nm wavelength was used to excite Opal 650 dye. Live imaging of cells was performed using a 25×/0.95 NA H20 objective with 75.7-nm pixel size and 1-μm z-spacing. Images of cryosections were taken with a 63x/1.2 NA H20 objective with 72-nm pixel size and 1-μm z-spacing, using a Zeiss Apotome 2 microscope and Zen Pro software.

### Immunogold Labeling

Immunogold labeling on cryosections of cross-sectioned optic nerves from c57Bl/6N mice at P75 and electron microscopy (in Figure 5) was performed as previously described (Werner et al., 2007; Lüders et al., 2017; Eichel et al., 2020). Primary antibodies were specific for CD59 (Invitrogen Cat# PA5-97565; 1:100).

### Analysis of Single-Cell RNA Sequencing Data

Single-cell RNA sequencing data of zebrafish oligodendrocyte lineage cells have been previously published (Marisca et al., 2020). Transcript per million (TPM) values in the publicly available dataset of gene expression in oligodendrocyte lineage cells (Accession number GSE132166; available at https://ki.se/en/mbb/oligointernode) were log-normalized with a scale factor of 10,000 as previously described (Marisca et al., 2020) to probe for expression of transcripts encoding known myelin proteins. The normalized abundance of myelin-related transcripts in the cells designated as oligodendrocyte precursor cells (OPC; clusters 1–4 in Marisca et al., 2020; *n* = 189 cells) and mature oligodendrocytes (mOL; cluster 5 in Marisca et al., 2020; *n* = 19 cells) was plotted. Precise *p*-values for comparing mRNA abundance in OPCs and mOLs by Welch’s *t*-test were *36k p* = 0.0004; *Cadm4 p* = 0.1324; *Cd59 p* = 0.0001; *Cd81 p* = 0.2202; *Cd82a p* = 0.1548; *Cfl p* = 0.0032; *Epb41l3a p* = 0.0299; *Lgi3 p* = 0.0017; *Mbpa p* = 0.0002; *Mpz p* = 0.0005; *Padi2 p* = 0.024, *Rhoab p* = 0.9608; *Sept7b p* = 0.0432; *Sept8b p* = 0.2665; *Sirt2 p* = 0.0003; *Slc12a2 p* = 0.0012; *Sox10 p* = 0.7829; *Cd9b, Cdc42, Cldn19, Cldnk, Mag, Mbpb, Ninj2, Nfasca, Plp1a, Plp1b, Tspan2a*, *Zw*i, *Olig2*, and *Myrf p* < 0.0001. Statistical test was performed in GraphPad Prism 8.

### Phylogenetic Analysis

Tblastn homology search of mRNA/EST databases was performed at blast.ncbi.nlm.nih.gov using CD59 or MPZ protein sequences from various species as queries. mRNA/EST sequences encoding CD59 or MPZ in selected species were retrieved. The longest available ORFs were translated using EditSeq for protein alignment using MegAlign with the algorithm ClustalW and protein weight matrix PAM250. Phylogenetic trees were constructed using Drawtree of the Phylip software package (Felsenstein, 1997) version 3.69 (Department of Genetics, University of Washington, Seattle) freely accessible at evolution.genetics.washington.edu/phylip/doc/drawtree.html.

### Deposition, Visualization, and Analysis of Data

The mass spectrometry proteomics data have been deposited to the ProteomeXchange Consortium via the PRIDE (Perez-Riverol et al., 2019) partner repository with dataset identifier PXD023037. Images were analyzed using Fiji (Schindelin et al., 2012), and figures and graphs were prepared in Microsoft Excel 2020, GraphPad Prism 8, and Adobe Illustrator CS6. Protein structure comparison was performed using Clustal Omega (EMBL EBI). Area-proportional Venn diagrams were prepared using BioVenn (Hulsen et al., 2008) at www.bio-venn.nl.

## Results

### Proteome Analysis of Myelin Purified From Zebrafish Brains

**FIGURE 1:**
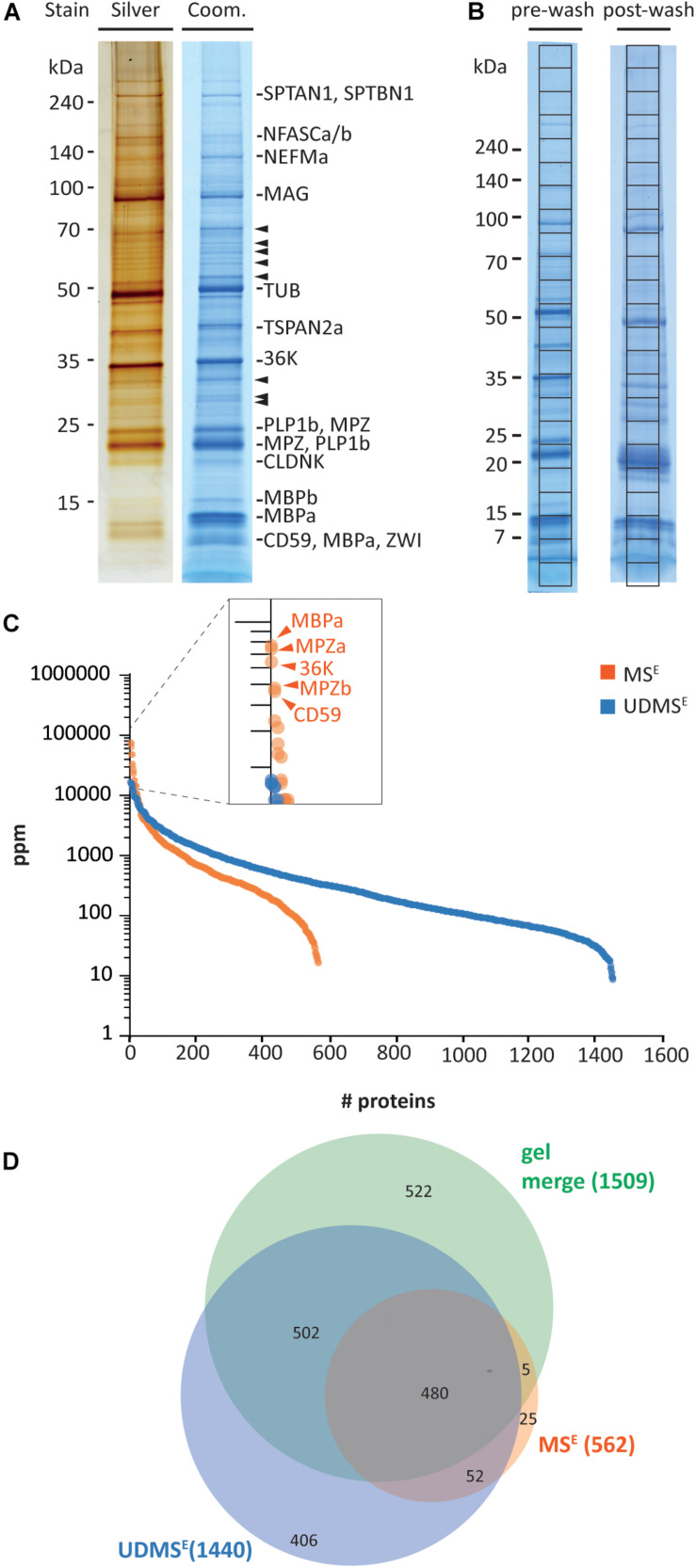
Proteome analysis of zebrafish myelin. **(A)** One-dimensional separation of myelin biochemically purified from brains and optic nerves of adult zebrafish. Myelin was separated by 1D SDS-PAGE on Tris-glycine gradient gels, and proteins were visualized by silver staining (0.5 μg load) or colloidal Coomassie (CBB250; 5 μg load). Bands are annotated that mainly comprise known myelin proteins according to mass spectrometric identification. Arrowheads indicate bands in which no known myelin proteins were identified. **(B)** Myelin separated on Tris-glycine gradient gels (5 μg load) before (pre-wash) or after (post-wash) an additional step of high-pH and high-salt conditions. The indicated grid divides each CBB250-stained lane into equally sized slices, which were excised for automated tryptic digest and LC-MS analysis, thereby, respectively, identifying 890 (pre-wash) and 1274 (post-wash) proteins (Supplementary Table 1). **(C)** Number and relative abundance of proteins identified and quantified in purified myelin by in-solution digestion and two data independent acquisition MS modes, MS^E^ and UDMS^E^. Note that MS^E^ (orange) identifies fewer proteins but provides a higher dynamic range for quantification of proteins in purified myelin compared to UDMS^E^. MS^E^ thus facilitates quantification of highly abundant myelin proteins. ppm, parts per million. **(D)** Venn diagram comparing the number of proteins identified in myelin by MS^E^, UDMS^E^, and gel-based approaches.

Aiming to systematically approach the protein composition of CNS myelin in a non-mammalian species, we reassessed a myelin-enriched lightweight membrane fraction purified from the brains and optic nerves of adult zebrafish. Purification of this fraction was previously reported (Nawaz et al., 2013); three pools of zebrafish brains and optic tracts were subjected to an established protocol of discontinuous sucrose density gradient centrifugation and osmotic shocks, resulting in biological triplicates (*n* = 3) used in the present analysis. By electron microscopy, the fraction virtually entirely contained multilamellar myelin sheaths (Supplementary Figure 1A). As a pilot experiment, we separated the myelin-enriched fraction on 1D SDS-PAGE gels followed by silver or colloidal Coomassie staining, thereby visualizing a considerable number of distinct bands (Figure 1A). When we excised Coomassie-stained bands and subjected them to tryptic in gel-digest and mass spectrometric protein identification, we found that the major bands comprised known myelin proteins (denoted in Figure 1A). Yet, several bands contained proteins not previously associated with myelin (arrowheads in Figure 1A). This pilot experiment thus supported our notion that the complexity of the protein composition of zebrafish myelin has not been fully uncovered yet.

To fill this gap, we performed systematic proteome analysis using both gel-based and gel-free methods. For simplicity, in the following, we use the terms “myelin” for the myelin-enriched biochemical fraction and “myelin-associated” for proteins identified in the fraction, irrespective if the localization was validated by independent approaches. Upon pre-fractionation of 5 μg of myelin on 8–16% Tris-glycine gradient gels (Figure 1B), slicing of the complete lanes into uniform pieces, and tryptic in gel-digest and peptide separation by liquid chromatography (LC) coupled to detection by mass spectrometry (LC-MS), we identified 890 proteins (labeled “pre-wash” in Figure 1B and Supplementary Table 1). Upon subjecting myelin to consecutive high-salt and high-pH washing/centrifugation cycles for enriching membrane proteins, the same procedure yielded 1,274 proteins (labeled “post-wash” in Figure 1B and Supplementary Table 1). When comparing the proteins identified in pre-wash versus post-wash myelin, we found considerable overlap. The two approaches combined yielded 1509 proteins, thus representing a comprehensive compendium of the zebrafish myelin proteome (Supplementary Table 1).

Considering that gel-based approaches often provide limited capabilities for mass spectrometric protein quantification, we also subjected myelin to an established gel-free workflow of solubilization, automated in-solution tryptic digest, peptide fractionation, and data-independent acquisition on a quadrupole time of flight mass spectrometer with ion mobility spectrometry option (Q-IMS-TOF type) (Jahn et al., 2020; Siems et al., 2020). These label-free methods termed MS^E^ and—when ion mobility-enhanced—UDMS^E^ allow simultaneous identification and quantification of myelin proteins. When subjecting zebrafish myelin to MS^E^ analysis, we identified and quantified 562 proteins (labeled in orange in Figure 1C and Supplementary Table 1) with a FDR of <1% at both the peptide and protein level and an average sequence coverage of 36.2%. Given that MS^E^ provides a high dynamic range of more than four orders of magnitude for quantification at the cost of proteome coverage, we consider the MS^E^ dataset a valid quantitative representation of the myelin proteome as the relative abundance even of the dominant proteins is determined correctly. When analyzing the myelin samples using the UDMS^E^ mode, in which ion mobility is utilized to further separate the peptides after chromatographic separation and before mass measurement, we identified and quantified 1440 proteins with an average sequence coverage of 32.6% (labeled in blue in Figure 1C and Supplementary Table 1). Thus, UDMS^E^ provides a deeper proteome coverage at the cost of dynamic range, which is limited to about three orders of magnitude. We consider the UDMS^E^ dataset a semi-quantitative inventory of the myelin proteome as it likely covers the vast majority of myelin-associated proteins—while underestimating the relative proportion of high-abundant but not of medium to low-abundant proteins. In the present label-free quantification approaches, this is indeed reflected by the identification of over twofold as many proteins by UDMS^E^ and the superior dynamic range of MS^E^ (Figure 1C), in line with previous observations on myelin preparations from mice (Jahn et al., 2020; Siems et al., 2020). When comparing the proteins identified by MS^E^, UDMS^E^, and gel-based approaches, we identified a total number of 1992 proteins in zebrafish myelin, among those 480 proteins by all approaches (Figure 1D).

### Relative Abundance of Myelin Proteins in the Zebrafish CNS

**FIGURE 2:**
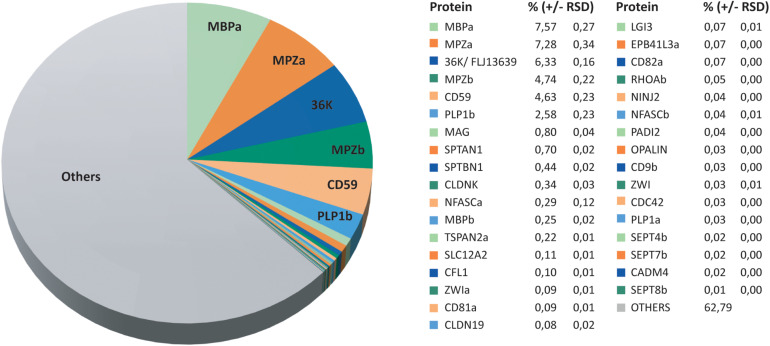
Relative abundance of CNS myelin proteins in zebrafish. Pie chart visualizing the MS^E^ dataset (Figure 1C and Supplementary Table 1). The relative protein abundance is given in percent with relative standard deviation (RSD). Note that known myelin proteins constitute about 37% of the total myelin protein, whereas proteins so far not associated with myelin constitute the remaining 63%.

Considering that quantification of the most abundant myelin proteins requires a high dynamic range (Jahn et al., 2020; Siems et al., 2020), we calculated the relative abundance of the 562 proteins identified in zebrafish myelin by MS^E^. According to this dataset, the most abundant zebrafish myelin proteins are myelin basic protein (MBPa), the Ig-CAM myelin protein zero (MPZa), the short-chain dehydrogenase 36K (also termed DHRS12), MPZb, and the complement inhibitor CD59, which respectively, constitute 7.6%, 7.3%, 6.3%, 4.7%, and 4.6% of the total myelin protein (Figure 2). When the abundance of protein paralogs is added together, MPZa/MPZb (Supplementary Figure 2) and MBPa/MBPb (Nawaz et al., 2013; Torvund-Jensen et al., 2018) are the most abundant constituents, comprising 12.0% and 7.8% of the total myelin protein, respectively. Besides the known zebrafish CNS myelin proteins MPZ (Brösamle and Halpern, 2002; Schweitzer et al., 2003; Bai et al., 2011), MBP (Li et al., 2007; Nawaz et al., 2013), and 36K (Morris et al., 2004; Nagarajan et al., 2020), CD59 was thus also identified as highly abundant. In addition, we identified and quantified multiple known myelin proteins at lower abundance, including proteolipids (PLP1a and PLP1b), claudins (CLDNK and CLDN19), Ig-CAMs (CADM4, CNTN1b, MAG, NFASCa, NFASCb, and NINJ2), tetraspanins (CD9b, CD81a, CD82a, and TSPAN2a), scaffolding proteins (EPB41L3a, SPTAN1, and SPTBN1), cytoskeletal proteins SEPT4b, SEPT7b, SEPT8b, CFL1, and PADI2), the ion transporter SLC12A2, and the teleost-specific zwilling proteins (ZWIa and ZWIb). The relative abundance of these proteins in myelin is shown in Figure 2. Together, by MS^E^, known myelin proteins constitute about 37% of the total zebrafish CNS myelin protein (Figure 2) while proteins not yet associated with myelin account for the remaining 63%. Categorization of proteins identified in the myelin-enriched fraction indicates only minor levels of contaminants from other cellular compartments (Supplementary Figure 1B), in accordance with the virtually exclusive multi-lamellar appearance of the fraction’s constituents by electron microscopy (Supplementary Figure 1A).

### Comparison to Related Datasets

Several recent studies have systematically assessed the abundance of proteins in myelin or of transcripts in cells of the oligodendrocyte lineage. To compare the zebrafish myelin proteome with such profiles, we correlated our quantitative proteome datasets (Supplementary Table 1) via gene name entries with available datasets.

**FIGURE 3:**
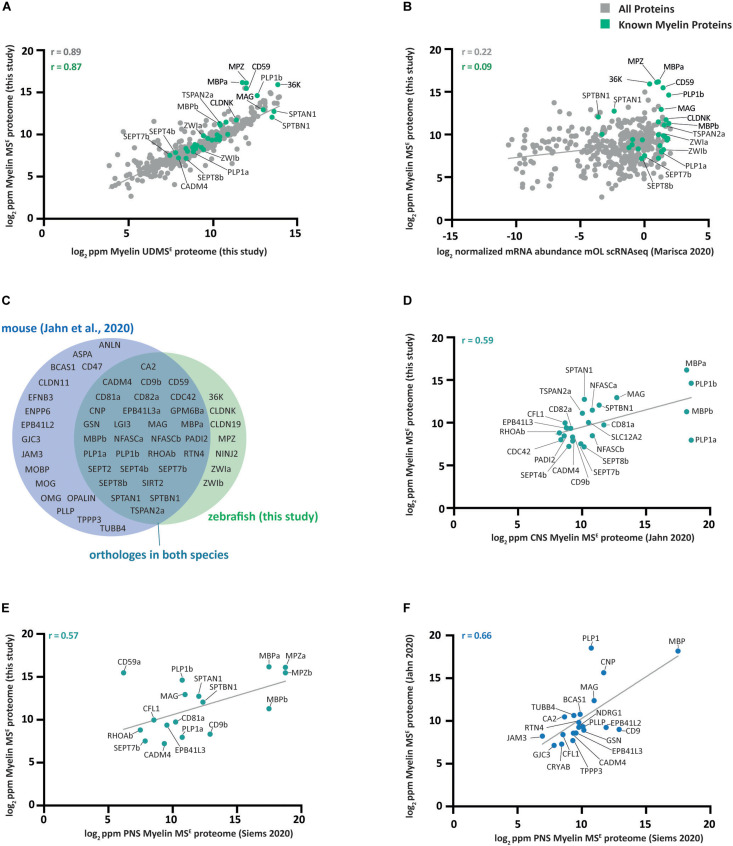
Comparison of the zebrafish myelin proteome with other datasets. **(A)** Scatter plot of the log_2_-transformed relative abundance of proteins identified in this study by MS^E^ in zebrafish CNS myelin against those identified by UDMS^E^. Data points representing known myelin proteins are labeled in green; other data points are in gray. The correlation coefficient was calculated for known myelin proteins (green) and for all proteins (gray) identified using both MS modes. The linear regression line is plotted for all proteins. ppm, parts per million. **(B)** Same as **(A)** but plotted against the scRNA-seq-based transcriptome of mature zebrafish oligodendrocytes (mOL) according to the normalized mean of all 19 cells in the mOL cluster according to prior assessment of oligodendrocyte lineage cells (Marisca et al., 2020). **(C)** Venn diagram comparing known myelin proteins identified in zebrafish CNS myelin in this study with those previously identified in CNS mouse myelin (Jahn et al., 2020). If a paralog has been reported in zebrafish, the protein name of the paralog is given. **(D)** Same as **(A)** but only known myelin proteins identified by MS^E^ in zebrafish myelin are plotted against the CNS myelin proteome of c57Bl/6N mice as previously assessed by the same mode (Jahn et al., 2020). Only proteins present in both datasets are plotted. **(E)** Same as **(D)** but known myelin proteins identified by MS^E^ in zebrafish CNS myelin are plotted against the mouse PNS myelin proteome as previously assessed by the same mode (Siems et al., 2020). **(F)** Same as **(D)** but known myelin proteins identified by MS^E^ in mouse CNS myelin mode (Jahn et al., 2020) are plotted against those identified by MS^E^ in mouse PNS myelin (Siems et al., 2020).

First, we plotted the myelin protein abundance profiles in the current MS^E^ and UDMS^E^ datasets (Supplementary Table 1) against each other (Figure 3A). As these datasets were established using the same samples and a similar workflow only differing in the MS measurement mode, it is not surprising that the datasets correlate well with a high correlation coefficient of 0.89. In line with the higher dynamic range of MS^E^ for quantification (Figure 1C), it was expected that the most abundant proteins MPZ, MBPa, CD59, and 36K were found to deviate most visibly from the linear regression line, confirming that they were under-quantified in UDMS^E^ due to ceiling effects occurring during mass spectrometric detection of ion mobility-separated peptides of exceptional abundance. We thus selected the MS^E^ dataset (Figures 1C, 2) as the myelin proteome reference for comparison with related datasets.

Next, we compared our MS^E^ dataset with previously established single-cell RNA sequencing (scRNAseq) data (Marisca et al., 2020) of oligodendrocyte lineage cells isolated from the zebrafish CNS at 5 dpf. For this comparison, we calculated the mean of the normalized mRNA abundance of all 19 cells in the cluster composed of mature oligodendrocytes (mOL). Comparing the myelin proteome with the oligodendrocyte transcriptome in zebrafish, we calculated a correlation coefficient of 0.22 (Figure 3B). Notwithstanding that differences in the age of animals, sample preparation and data analysis probably affect the degree of correlation, it is noteworthy that an approximately similar correlation coefficient was previously found when comparing the myelin proteome with the oligodendrocyte transcriptome in mice (Jahn et al., 2020).

We then compared CNS myelin proteins between zebrafish and mice, using the present data and a previously established mouse myelin proteome dataset (Jahn et al., 2020). We focused on known myelin proteins (Figure 2), as for several proteins readily identified in zebrafish myelin no orthologs were found in mouse myelin, including 36K, CLDN19, CLDNK, ZWIa, and ZWIb (Figure 3C). *Vice versa*, for numerous myelin proteins previously established in mice, no ortholog was identified in zebrafish myelin, including claudin 11 (CLDN11), myelin oligodendrocyte basic protein (MOBP), myelin oligodendrocyte protein (MOG), oligodendrocytic myelin paranodal and inner loop protein (OPALIN), plasmolipin (PLLP), and anillin (ANLN) (Figure 3C). Together, this indicates heterogeneity of the myelin proteome. Yet, a surprisingly large number of proteins displayed orthologs in myelin of both species. Importantly, this was not limited to classical structural myelin proteins PLP1/PLP1a/PLP1b and MBP/MBPa/MBPb. Orthologs in myelin of both species were also identified for Ig-CAMs (MAG, CADM4, and NFASC/NFASCa/NFASCb), tetraspanins (CD9/CD9b, CD81/CD81a, CD81/CD82a, and TSPAN2/TSPAN2a), myelin septins (SEPT4/SEPT4b, SEPT7/SEPT7b, and SEPT8/SEPT8b), signaling proteins (CDC42 and RHOA/RHOAb), the ion transporter SLC12A2, as well as the enzymes sirtuin 2 (SIRT2), carbonic anhydrase 2 (CA2), and peptidyl arginine deiminase PADI2 (Figure 3C). Comparing all proteins with orthologs in myelin of both species resulted in a comparatively high correlation coefficient of 0.59 (Figure 3D), implying that myelin protein composition is partially conserved between zebrafish and mice. Yet, we note that the correlation coefficient is roughly similar when comparing known myelin proteins in the zebrafish CNS myelin proteome with those in the PNS of mice (Figure 3E). A similar comparison between known myelin proteins in the PNS and CNS of mice yielded a somewhat higher correlation coefficient of 0.66 (Figure 3F), implying that the abundance profile of myelin proteins in the CNS of mice is closer related to that in the mouse PNS than to that in the zebrafish CNS.

### Expression of Myelin Proteins in the Oligodendrocyte Lineage

**FIGURE 4:**
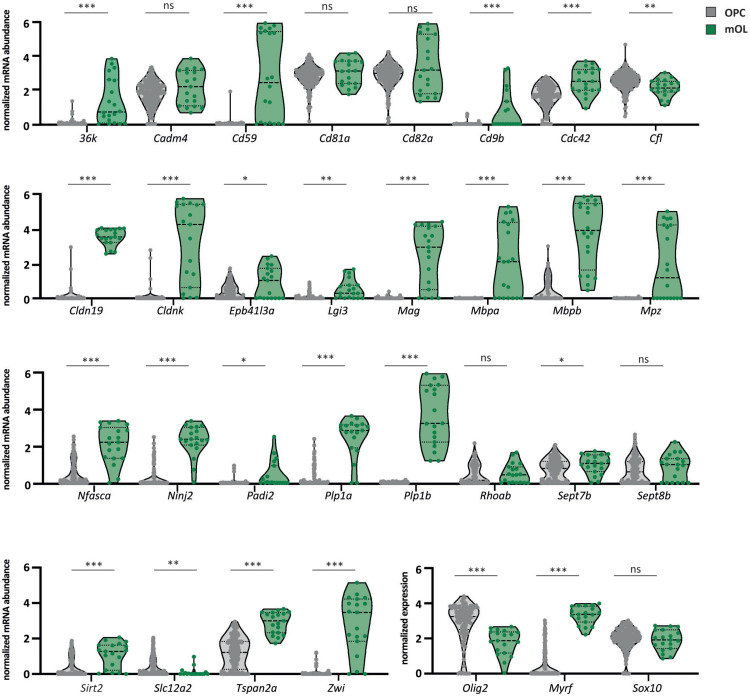
Expression of myelin-related mRNAs in OPCs and mature oligodendrocytes. Violin plots comparing the relative abundance of selected myelin-related transcripts in OPCs and mature oligodendrocytes according to a previously established scRNAseq dataset (Marisca et al., 2020). Data are given as normalized log-transformed mRNA abundance in transcripts per million (TPM) in OPCs (gray) versus mature oligodendrocytes (mOL) (green). Median with interquartile ranges; *n* = 189 OPCs (combined clusters 1–4 in Marisca et al., 2020) versus *n* = 19 mOL (cluster 5 in Marisca et al., 2020); ^∗^*p* < 0.05, ^∗∗^*p* < 0.01, ^∗∗∗^*p* < 0.001 by Welch’s *t*-test. For precise *p*-values, see section “Materials and Methods.” The mRNAs for oligodendrocyte transcription factor 2 (*olig2*), myelin regulatory factor (*myrf*), and SRY-box transcription factor 10 (*sox10*) serve as markers. ns, not significant.

To assess the abundance profiles of myelin-related transcripts during maturation of zebrafish oligodendrocytes, we filtered a previously published scRNAseq dataset for selected mRNAs corresponding to our proteome dataset and plotted their normalized abundance levels in oligodendrocyte precursor cells (clusters OPC 1–4 in Marisca et al., 2020) and mature oligodendrocytes (cluster mOL in Marisca et al., 2020). Notwithstanding that the different numbers of OPCs and mOL for which transcript abundance profiles are available may affect the biological inference, most myelin-related transcripts displayed a markedly increased abundance in mature oligodendrocytes compared to OPCs. Notably, this includes *Mpza*, *Mbpa*, *Mbpb*, *36k*, *Plp1a*, *Plp1b*, *Mag*, *Cldnk*, *Cldn19, Sirt2*, and *Tspan2a* (Figure 4), implying that their expression coincides with myelination. Yet, not all cells in the mature oligodendrocyte cluster displayed high abundance of all myelin-related transcripts, implying heterogeneity of oligodendroglial mRNA expression profiles. We also note that the abundance of transcripts encoding myelin septins (*Sept7b and Sept8b*), as well as *Cd81a* and *Cd82a*, were approximately similar in OPCs and mature oligodendrocytes, suggesting that their expression does not depend on the oligodendroglial maturation stage.

### Expression and Subcellular Localization of CD59 in Myelinating Cells

**FIGURE 5:**
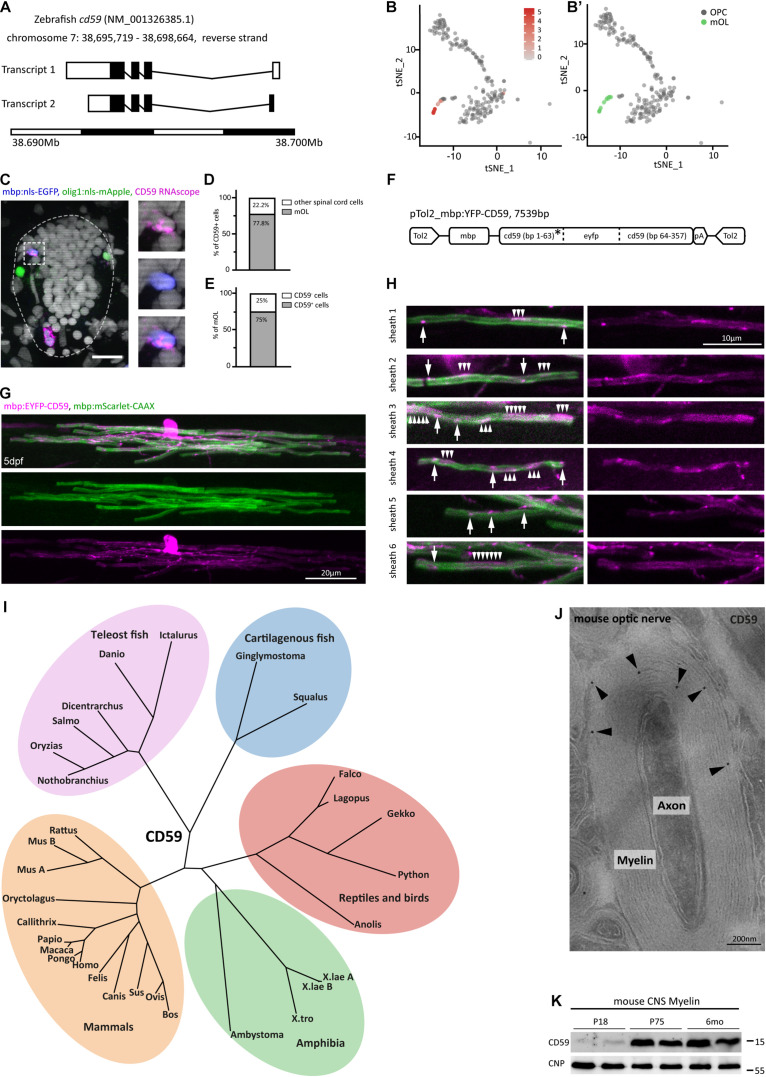
Expression and subcellular localization of CD59 in the zebrafish spinal cord. **(A)** Gene structure of zebrafish *cd59*. Filled boxes indicate the open reading frame. **(B)** tSNE plot showing cells displaying high *cd59* transcript abundance (labeled in red) by scRNAseq (Marisca et al., 2020). Cells with low or absent *cd59* expression are labeled in gray. **(B′)** Oligodendrocyte lineage cluster layout for the tSNE plot in **(B)** with the 19 cells in the mature oligodendrocyte cluster (mOL; green) and the combined OPC clusters (gray). Note the overlap between high *cd59* expression and the mature oligodendrocyte cluster. **(C)** Example micrographs of RNAscope *in situ* hybridization detecting *cd59* transcripts in transverse spinal cord sections of Tg(olig1:nls-mApple × mbp:nlsGFP) zebrafish larvae at 5 dpf (scale bar, 10 μm). Magnified images to the right show a *Cd59* + (purple) mbp:nls-EGFP (blue) mature oligodendrocyte (individual channels and merge). Seventy-two transverse sections of four animals were taken and imaged for quantification in **(D,E)**. No *Cd59* + cells were found positive for olig1:nls-mApple (green) representing OPCs. **(D)** Quantification of the percentage of *cd59* RNAscope-positive cells that were mature oligodendrocytes (mOL) according to expression of the mbp:nlsEGFP transgene. *n* = 27 oligodendrocytes from 72 transverse sections taken along the entire spinal cord (anterior, mid-trunk, posterior) of four individual animals were quantified. **(E)** Quantification of the percentage of mbp:nlsGFP transgene positive mature oligodendrocytes (mOL) expressing *cd59* transcripts to an abundance identified by RNAscope *in situ* hybridization. *n* = 28 cells from four individual animals were quantified. **(F)** Schematic of plasmid design for expression of a CD59 reporter construct in mature oligodendrocytes. The *eyfp* open reading frame was inserted after the sequence encoding the CD59 signal peptide (SP, labeled with an asterisk). **(G)** Example image of an individual mature oligodendrocyte at 4 dpf co-expressing membrane targeted mScarlet-CAAX and EYFP-CD59. Scale bar, 20 μm. **(H)** Examples of individual myelin sheaths in micrographs as in **(G)**. Arrows point at discrete CD59 puncta, and arrowheads point to broader patches of CD59 localization. Scale bar, 10 μm. **(I)** Phylogeny of CD59 in an unrooted phylogenetic tree. All phylogenetic relationships are in agreement with the hypothesis that CD59 emerged at the root of vertebrates and was retained in all vertebrate groups. Two CD59 paralogs (CD59a and CD59b) exist in mice. There is no evidence of CD59 paralogs in teleost fish. **(J)** Immunodetection of CD59 (10-nm gold particles; black arrowheads) on cross-sectioned optic nerves of mice at postnatal day 75 (P75). Micrograph representative of 50 axon/myelin units in *n* = 2 biological replicates. Note that CD59 is mainly detected in compact and abaxonal myelin. Scale bar, 200 nm. **(K)** Immunoblot analysis of myelin biochemically purified from c57Bl/6N mouse brains at ages P18, P75, and 6 months using antibodies specific for CD59. Blot shows *n* = 2 biological replicates per age. CNP was detected as control.

Considering that MPZ, MBP, and 36K were already previously recognized as abundant myelin proteins in the zebrafish CNS, we focused on CD59, the fourth-most abundant protein in our MS^E^ dataset (Figure 2). According to scRNAseq (Marisca et al., 2020), expression of the *cd59* gene (Figure 5A) was largely confined to the cluster comprising mature oligodendrocytes and absent from OPCs (Figures 4 and 5B). Indeed, an RNAscope *in situ* hybridization probe for *cd59* labeled discrete cells in the spinal cord of zebrafish (purple in Figure 5C), the large majority of which were identified as mature oligodendrocytes (Figure 5D) by transgenic expression of nuclear-targeted EGFP under control of the *mbp* gene regulatory elements (blue in Figure 5C). Notably, the majority of transgenically labeled mature oligodendrocytes were positive for the *cd59* RNAscope probe (Figure 5E). OPCs identified by transgenic expression of nuclear-targeted mApple under control of the *olig1* promoter (green in Figure 5C) were not labeled by the *cd59* RNAscope probe. Together, these data show that *cd59* expression is largely restricted to mature oligodendrocytes in the zebrafish CNS.

To assess the localization of CD59 in zebrafish oligodendrocytes, we microinjected mbp:EYFP-CD59 and mbp:mScarlet-CAAX reporter constructs into wild-type zebrafish, thereby obtaining sparse labeling of the myelin membrane and CD59 in individual oligodendrocytes (Figure 5F). Confocal imaging of labeled cells showed transgenically expressed EYFP-CD59 fusion protein localizing to both oligodendrocyte cell bodies and myelin sheaths (Figures 5G,H). Within individual internodes, the CD59 reporter was not diffusely localized throughout the myelin sheath but was restricted to discrete patches and punctae along the sheath (Figure 5H). This implies that CD59 expressed in oligodendrocytes is indeed incorporated into myelin sheaths.

Phylogenetic analysis of CD59 sequences in 29 species yielded a phylogenetic tree that largely reflects the known relationships among vertebrate groups (Figure 5I). Yet, we did not find evidence of CD59 paralogs in zebrafish or other teleost species, implying that only one *cd59* paralog was retained after genome duplication at the root of teleost fish. On the other hand, two closely related CD59 paralogs exist in mice, suggesting clade-specific duplication of the *Cd59* gene. Considering that CD59 is sufficiently well conserved across vertebrate evolution to allow assessment of its expression in mammals, we applied commercially available antibodies against CD59 coupled to gold particles to cross-sectioned optic nerves of mice. Indeed, by cryo-immuno electron microscopy, CD59 labeling was detected in compact and abaxonal myelin (Figure 5J). By immunoblotting, CD59 was readily detected in myelin purified from the brains of c57Bl/6N mice both at the age of postnatal day 75 (P75) and at 6 months (Figure 5K). It was interesting to note that CD59 was virtually undetectable in myelin purified from mouse brains at P18, implying a maturation-dependent increase of its abundance in mouse myelin. Together, these results establish CD59 as a constituent of CNS myelin in both zebrafish and mice, notwithstanding its comparatively low abundance in the latter (Jahn et al., 2020).

## Discussion

We have used gel-based and gel-free proteomic approaches to investigate the protein composition of myelin purified from the brains and optic nerves of adult zebrafish. Deep proteome coverage including the identification of many low-abundant constituents has yielded the largest compendium of zebrafish CNS myelin proteins thus far. Importantly, the combination of tryptic digest of proteins in solution and acquisition of mass spectrometric data in the MS^E^ mode provides a high dynamic range of four orders of magnitude and thus allows correct quantification of the exceptionally abundant proteins that dominate the myelin sheath. Comparing the MS^E^ dataset with previously established scRNAseq-based mRNA abundance profiles of zebrafish oligodendrocytes (Marisca et al., 2020), we found that most myelin-related transcripts display a considerable increase of their abundance at the transition from the OPC to the mature oligodendrocyte stage, implying co-regulated expression.

Our mass spectrometric quantification confirms that the most abundant constituent of zebrafish CNS myelin is MPZ, a cell adhesion protein comprising one immunoglobulin-like domain. Indeed, this domain mediates adhesion and compaction of the extracellular surfaces of adjacent myelin membranes in both mammals (D’Urso et al., 1990; Filbin et al., 1990; Giese et al., 1992) and fish (Lanwert and Jeserich, 2001). It has long been known that MPZ is an abundant myelin protein in both the PNS and the CNS of fish, while at the fish-to-tetrapod transition, MPZ was replaced by PLP as the major CNS myelin protein (Franz et al., 1981; Waehneldt et al., 1986; Kirschner et al., 1989; Yoshida and Colman, 1996; Lüders et al., 2019). Two highly abundant MPZ paralogs exist in zebrafish myelin according to mass spectrometric identification. Indeed, their identification by two to three unique peptides each allows a very high level of confidence. Moreover, both paralogs are supported by entries in the ENSEMBL database and several mRNA/EST entries, notwithstanding that only one *mpz* gene has been annotated in the ZFIN database by the time of writing. While the amino acid sequences of the paralogs differ only in a few distinct amino acids, they are identical in the transmembrane domain, extracellular glycosylation site, and two extracellular cysteines that form the disulfide bridge that structures the extracellular domain (Zhang and Filbin, 1994; Raasakka et al., 2019). It is thus presently speculative if the MPZ paralogs differ with respect to function or spatiotemporal expression. It is of note that the existence of two MPZ variants in several teleost species was already reported in the 1970s. Termed IP1 and IP2 at that time, they were often thought to represent splice isoforms (Franz et al., 1981; Jeserich, 1983; Waehneldt et al., 1986). Yet, our phylogenetic analysis of sequences available in public databases supports the existence of MPZ paralogs in (at least) three families of teleost fish, namely, Danionidae, Cyprinidae, and Salmonidae. On the other hand, we found no evidence for MPZ paralogs in the Neoteleostei clade. Together, this implies that two *mpz* paralogs emerged coinciding with the whole genome duplication at the root of teleosts and that one paralog was subsequently lost in a subgroup of teleostei, probably at the root of the Neoteleostei clade.

The second-most abundant zebrafish myelin protein is MBP, a cytoplasmic protein that displays high affinity to the negatively charged headgroups of membrane phospholipids (Musse et al., 2008; Nawaz et al., 2009, 2013), thereby allowing the close proximity of the intracellular membrane surfaces (Aggarwal et al., 2013). Indeed, MBP is rate-limiting for CNS myelination in mice (Roach et al., 1985; Popko et al., 1987; Readhead et al., 1987). Similar to MPZ, two MBP paralogs were identified in zebrafish myelin (MBPa and MBPb). MBPa is considerably more abundant in zebrafish myelin than MBPb, their spatiotemporal expression differs, and one distinct small MBPb splice isoform does not associate with the plasma membrane (Nawaz et al., 2013; Torvund-Jensen et al., 2018). Together, the maintenance of two MBP paralogs in teleost fish may reflect functional specialization.

36K, the third-most abundant zebrafish myelin protein, was also recognized early in several teleost fish species (Franz et al., 1981; Jeserich, 1983; Waehneldt et al., 1986; Kirschner et al., 1989; Moll et al., 2003) and subsequently identified by mass spectrometric methods as a short-chain dehydrogenase/reductase (DHRS12; Morris et al., 2004). More recently, 36K was found to regulate oligodendroglial Notch signaling and myelin lipid composition in zebrafish (Nagarajan et al., 2020). While an ortholog exists in mammals, we are not aware of evidence for its incorporation into mammalian myelin (Jahn et al., 2020; Siems et al., 2020).

Constituting over 4% of the total zebrafish myelin protein, we identified CD59 as the fourth-most abundant zebrafish myelin protein. Phylogenetic analysis did not yield evidence for *cd59* paralogs in teleosts but, remarkably, in mice. Similar to most other myelin-related transcripts, *cd59* mRNA abundance increases strongly at the transition from OPCs to mature oligodendrocytes, probably in a subpopulation. We note that CD59 has not yet been reported as an abundant fish myelin constituent. Considering that independent validation is required for a protein identified in the myelin-enriched fraction to be considered a genuine myelin protein, we imaged zebrafish expressing an EYFP-CD59 fusion protein in mature oligodendrocytes. Indeed, the fusion protein localized to myelin sheaths. Together, expression of *cd59* in mature oligodendrocytes according to scRNAseq and *in situ* hybridization, as well as the incorporation of CD59 into CNS myelin in transgenic zebrafish, circumstantially support the finding of CD59 as a major myelin protein in zebrafish. CD59 is also a myelin constituent in both the peripheral (Erne et al., 2002) and central nervous system (this paper) of mice, though of comparatively low abundance (Jahn et al., 2020; Siems et al., 2020).

The level of CD59 expression has been associated with immunomodulatory functions of mammalian oligodendrocytes as well as their susceptibility to complement-mediated lysis (Koski et al., 1996; Scolding et al., 1998; Ramaglia et al., 2009; Zeis et al., 2016). Indeed, experimental rodents lacking CD59 display enhanced demyelination in both experimental autoimmune encephalomyelitis (Mead et al., 2004) and the Aquaporin 4-IgG injection model of neuromyelitis optica (Zhang and Verkman, 2014; Yao and Verkman, 2017), in agreement with the view that CD59-expression protects against complement-mediated degeneration (Piddlesden and Morgan, 1993; Kolev et al., 2010). We thus speculate that the comparatively high abundance of CD59 in zebrafish myelin reflects relevance in inhibiting complement-dependent demyelination. However, further studies will be required to resolve the precise role of CD59 for myelin sheaths in the healthy and diseased nervous system. Interestingly, recent studies using both zebrafish and mice have revealed that individual myelin sheaths can be pruned by microglia, which may represent a form of myelin plasticity (Hughes and Appel, 2020; Djannatian et al., 2021). It is tempting to speculate that individual myelin sheaths may be susceptible to elimination if displaying a comparatively low abundance of CD59 or, *vice versa*, may be prevented from pruning by accumulating a high level of CD59. In this respect, it is interesting to note that the abundance of CD59 in myelin purified from mouse brains is low at an early developmental stage representing myelin biogenesis and increases coinciding with myelin maturation. Indeed, the observed heterogeneity of zebrafish mOL with respect to *cd59* mRNA expression may reflect the existence of newly formed and advanced mOL subpopulations. It will be interesting to address the functional relevance of this phenomenon and possible age-dependent changes in future studies.

As exemplified by CD59, the relative abundance of a myelin protein can differ markedly between zebrafish and mice, and some proteins such as MPZ and 36K are even CNS myelin constituents in zebrafish but not in mice. *Vice versa*, no ortholog was identified in zebrafish myelin for several classical mammalian myelin proteins including MOBP, MOG, CLDN11, and PLLP. Together, this indicates evolutionary heterogeneity of the CNS myelin proteome. On the other hand, the zebrafish myelin proteome comprised orthologs of a surprisingly large number of myelin proteins originally identified in rodents. Not unexpectedly, this includes structural myelin proteins such as PLP (PLP1a and PLP1b) (Schweitzer et al., 2006; Möbius et al., 2008), MBP (MBPa and MBPb) (Nawaz et al., 2013), and septins (SEPT4b, SEPT7b, and SEPT8b), which form a stabilizing filament in CNS myelin that prevents pathological myelin outfoldings at least in mice (Patzig et al., 2016a; Erwig et al., 2019b). Myelin-associated cell adhesion molecules with Ig-like domains (CNTN1b, MAG, NFASCb, and CADM4) (Haenisch et al., 2005; Djannatian et al., 2019; Elazar et al., 2019a, b; Klingseisen et al., 2019) were also identified as myelin constituents in both zebrafish and mice, indicating that major molecules that mediate axo-myelinic interactions are conserved between teleost fish and mammals, at least in part. Intriguingly, several enzymes enriched in mouse myelin (CNP, SIRT2, and PADI2) (Werner et al., 2007; Edgar et al., 2009; Falcão et al., 2019) were also identified (as orthologs) in zebrafish myelin, implying functional relevance beyond mammals. Finally, the zebrafish myelin proteome comprises multiple tetraspanins (CD9b, CD81a, CD82a, and TSPAN2a), of which orthologs were previously reported in rodent myelin (Terada et al., 2002; Ishibashi et al., 2004; Mela and Goldman, 2009; De Monasterio-Schrader et al., 2013). Considering their immunomodulatory functions (Zeis et al., 2016), the presence of these proteins in myelin of species as distinct as zebrafish and mice may reflect the existence of a functional core myelin proteome in maintaining a healthy nervous system. We thus believe that further exploitation of the present myelin proteome resource is promising toward better understanding molecular and functional heterogeneity and similarities of myelin across the vertebrate clade.

## Data Availability Statement

The mass spectrometry proteomics data have been deposited to the ProteomeXchange Consortium via the PRIDE (Perez-Riverol et al., 2019) partner repository with dataset identifier PXD023037.

## Ethics Statement

Ethical review and approval was not required for the animal study because for the procedure of sacrificing vertebrates for subsequent preparation of tissue, all regulations given in the German Animal Protection Law (TierSchG §4) are followed. Since sacrificing of vertebrates is not an experiment on animals according to §7 Abs. 2 Satz 3 TierSchG, no specific authorization or notification is required for the present work. Breeding of transgenic zebrafish at University of Edinburgh was carried out in accordance with United Kingdom Home Office regulations under the project license PP5258250.

## Author Contributions

SS, RJ, DH, WM, and LH performed the experiments. SS, OJ, LH, and HW analyzed the data and performed statistical analysis. OJ, TC, and HW designed and directed the study. HW conceived the study and wrote the manuscript with major contributions by SS, OJ, and TC. All authors contributed to revising the manuscript and approved the submitted version.

## Conflict of Interest

The authors declare that the research was conducted in the absence of any commercial or financial relationships that could be construed as a potential conflict of interest.

## Supplementary Material

Supplementary Figure 1 Assessment of the myelin-enriched fraction. **(A)** Representative electron micrograph (EM) of the light-weight myelin-enriched membrane fraction purified from the brains and optic nerves of adult zebrafish. Myelin sheaths are identified by their typical multimembrane structure; cellular compartments other than myelin were virtually undetectable. **(B)** Pie chart visualizing the MS^E^ dataset (Figure 1C and Supplementary Table 1). The relative protein abundance is given in percent. Note that known myelin proteins constitute about 37% of the total myelin protein as specified in Figure 2. Mitochondria as represented by OXPHOS (oxidative phosphorylation) proteins appear as the most common contaminants of the myelin fraction from zebrafish brains, comprising about 0.7% of the total protein. Nuclear proteins represented by histones comprised < 0.1%. Proteins of the extracellular matrix (ECM) were not identified, as judged by the absence of its constituents including laminins, collagens, fibronectin, elastin, perlecan, dystroglycan, and fibrillin in the MS^E^ dataset (Supplementary Table 1).

Supplementary Figure 2 Phylogeny of myelin protein zero (MPZ). **(A)** Sequence alignment of MPZ paralogs (MPZa and MPZb) identified in zebrafish myelin. Predicted signal peptide is in italics, extracellular cysteine residues likely forming disulfide bridge are in magenta, N-glycosylation site (motif NGT) is in orange and transmembrane domain is in blue. Mass spectrometrically identified peptides are underlined (dotted line, MS^E^ only; single line, UDMS^E^ only; double line, both MS^E^ and UDMS^E^) and shaded in gray when also confirmed by gel-based proteomic data. Note that 2–3 unique peptides per paralog (green and red) were identified that discriminate the paralogs. Three individual amino acid differences in otherwise homologous peptide sequences are highlighted in yellow to improve visibility. **(B)** Unrooted phylogenetic tree. All phylogenetic relationships are in agreement with the hypothesis that two MPZ paralogs (MPZa, MPZb) emerged coinciding with the whole genome duplication at the root of teleosts which were both retained in the Danionidae, Cyprinidae and Salmonidae families while one paralog was lost at the root of the Neoteleostei clade.

Supplementary Table 1 Compendium of the zebrafish myelin proteome. Sheet 1: 1D gel separation of proteins followed by in-gel digestion and LC-MS analysis (890 proteins). Sheet 2: 1D gel separation of proteins after membrane wash followed by in-gel digestion and LC-MS analysis (1,274 proteins). Sheet 3: In-solution digestion of proteins and label-free quantification by MS^E^ (562 proteins). Sheet 4: In-solution digestion of proteins and label-free quantification by UDMS^E^ (1,440 proteins). Sheet 5: Compendium providing information on the approach by which a myelin-associated protein was identified (1,992 proteins).
