# Supplementary figures and images for: Proteome Profile of Myelin in the Zebrafish Brain
Source: Front Cell Dev Biol. 2021 Apr 8;9:640169. doi: 10.3389/fcell.2021.640169 (PMC8060510; doi:10.3389/fcell.2021.640169)

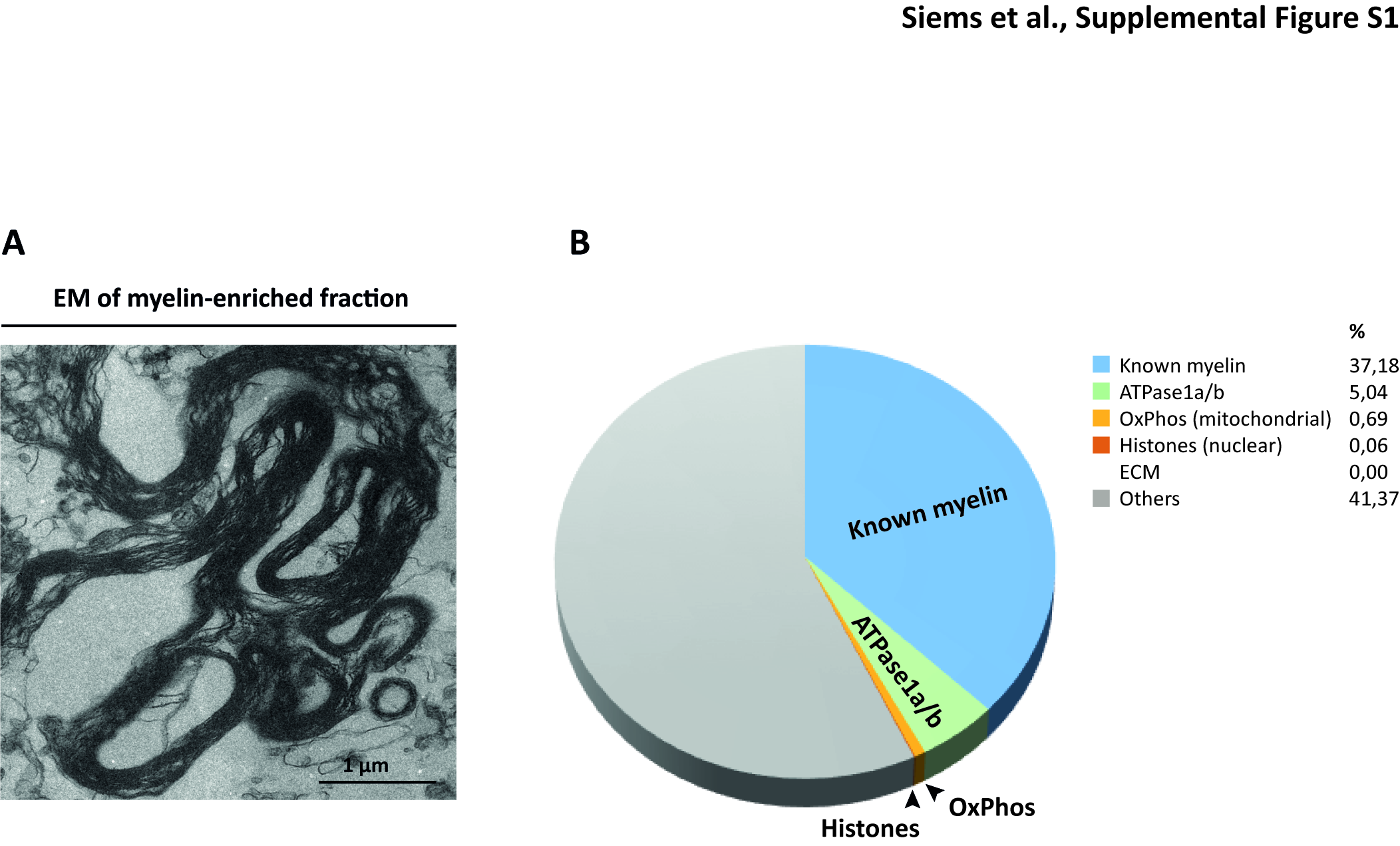

Supplement: Supplementary Figure 1 — Assessment of the myelin-enriched fraction. (A) Representative electron micrograph (EM) of the light-weight myelin-enriched membrane fraction purified from the brains and optic nerves of adult zebrafish. Myelin sheaths are identified by their typical multimembrane structure; cellular compartments other than myelin were virtually undetectable. (B) Pie chart visualizing the MSE dataset (Figure 1C and Supplementary Table 1). The relative protein abundance is given in percent. Note that known myelin proteins constitute about 37% of the total myelin protein as specified in Figure 2. Mitochondria as represented by OXPHOS (oxidative phosphorylation) proteins appear as the most common contaminants of the myelin fraction from zebrafish brains, comprising about 0.7% of the total protein. Nuclear proteins represented by histones comprised < 0.1%. Proteins of the extracellular matrix (ECM) were not identified, as judged by the absence of its constituents including laminins, collagens, fibronectin, elastin, perlecan, dystroglycan, and fibrillin in the MSE dataset (Supplementary Table 1). [file Image_1.TIF]

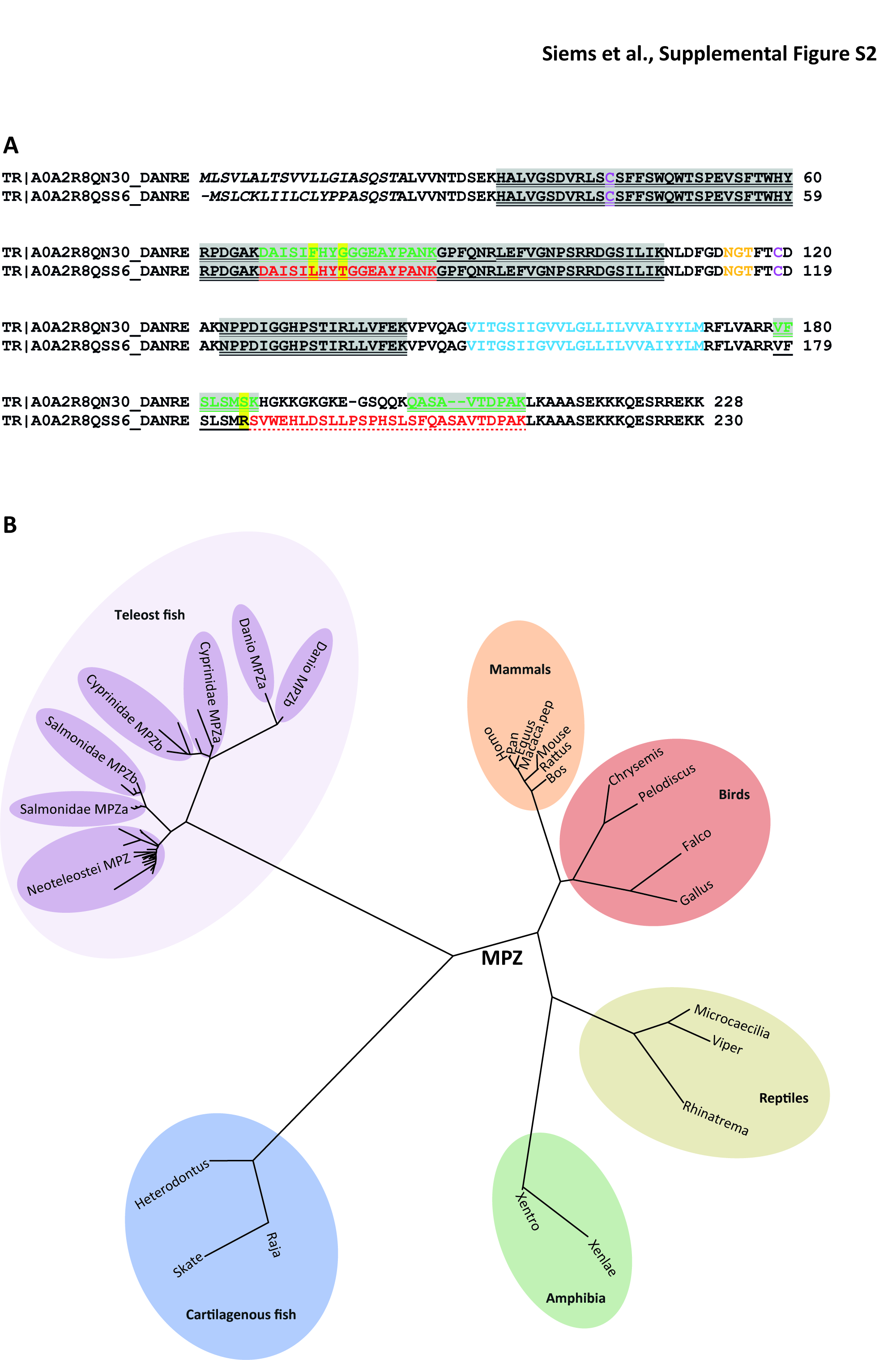

Supplement: Supplementary Figure 2 — Phylogeny of myelin protein zero (MPZ). (A) Sequence alignment of MPZ paralogs (MPZa and MPZb) identified in zebrafish myelin. Predicted signal peptide is in italics, extracellular cysteine residues likely forming disulfide bridge are in magenta, N-glycosylation site (motif NGT) is in orange and transmembrane domain is in blue. Mass spectrometrically identified peptides are underlined (dotted line, MSE only; single line, UDMSE only; double line, both MSE and UDMSE) and shaded in gray when also confirmed by gel-based proteomic data. Note that 2–3 unique peptides per paralog (green and red) were identified that discriminate the paralogs. Three individual amino acid differences in otherwise homologous peptide sequences are highlighted in yellow to improve visibility. (B) Unrooted phylogenetic tree. All phylogenetic relationships are in agreement with the hypothesis that two MPZ paralogs (MPZa, MPZb) emerged coinciding with the whole genome duplication at the root of teleosts which were both retained in the Danionidae, Cyprinidae and Salmonidae families while one paralog was lost at the root of the Neoteleostei clade. [file Image_2.TIF]
